# Editorial for the Special Issue on Optofluidic Devices and Applications

**DOI:** 10.3390/mi11100884

**Published:** 2020-09-23

**Authors:** Francisco Yubero, Fernando Lahoz

**Affiliations:** 1 Instituto de Ciencia de Materiales de Sevilla (CSIC–University Sevilla), 41092 Sevilla, Spain; 2 Departamento de Física, IUdEA, Universidad de La Laguna, 38200 Santa Cruz de Tenerife, Spain

Optofluidic devices are of high scientific and industrial interest in chemistry, biology, material science, pharmacy, and medicine. In recent years, they have experienced a strong development because of impressive achievements in the synergistic combination of photonics and micro- and nano-fluidics. Thus, sensing and/or lasing platforms showing unprecedented sensitivities in extremely small analyte volumes, and allowing real-time analysis within a lab-on-a-chip approach, have been developed. They are based on the interaction of fluids with evanescent waves induced at the surface of metallic or photonic structures, on the implementation of microcavities to induce optical resonances in the fluid medium or on other interactions of the microfluidic systems with light. In this context, a large variety of optofluidic devices have emerged, covering topics such as cells manipulation, microfabrication, water purification, energy production, catalytic reactions, microparticle sorting, micro-imaging, or bio-sensing. Moreover, integration of these optofluidic devices in larger electro-optic platforms represents a highly valuable improvement towards advanced applications, such as those based on surface plasmon resonances, already in the market.

In this Special Issue on Optofluidic Devices and Applications, we include 10 papers, covering different aspects related to water quality monitoring (1) and purification (4), structural stability of nanohole array-based devices (2), hydrodynamical focusing (3), fluorescence-based thermometry (5), electrofluidics (6), electrowetting (7) and optofluidic manipulation (8). Additionally, there are two interesting review papers on opto-electrokinetic-based manipulation and fabrication (9) and portable optofluidic systems for ocean monitoring (10).

In particular, Zuo et al. describe an optofluidic device for precise real-time detection of dissolved oxygen based on the plasmon resonant shift of silver nanoprisms with potential application in biomedical or water sensing fields [1]. Bdour et al. present an investigation of the deflection and structural stability of nanohole array-based optofluidic sensors operating in flow-through mode [2]. Hamilton et al. describe a study where 3D hydrodynamic focusing was implemented in 10 μm scale microchannel cross-sections made with a single sacrificial layer, which, implemented in optofluidic sensors, enable higher detection sensitivity and sample specificity [3]. Li et al. report a paper-based photocatalyst immobilization method with enhanced purification efficiency that solves the so-called “coffee ring effect” that occurs on the substrate during solvent evaporation, resulting in the aggregation of the photocatalysts [4]. Ghifari et al. describe a proof of concept of an optofluidic method based on dye-doped ZnO microcapsules for high throughput fluorescence-based thermometry, which enables the measure of temperature inside optofluidic microsystems at the millisecond time scale [5]. Deng et al. report on two kinds of electro-fluidic dyes based on anthraquinone and azo pyrazolone, including their synthesis, structure characterization, and application properties [6]. Yi et al. present a study related to the aperture ratio improvement by optimizing the voltage driving waveform for electrowetting displays [7]. Winskas et al. demonstrate a new bi-metallic substrate that allows micro-scale optofluidic manipulation controlled by an external laser power [8]. Liang et al. present a variety of differently structured optoelectrokinetic chips, discussing how they are fabricated and the ways in which they work. They also provide a summary of the current challenges of optoelectrokinetics and their future prospects [9]. Finally, Wang et al. describe the applications of optofluidic platforms on autonomous and in situ ocean environmental monitoring, with an emphasis on their principles, sensing properties, advantages, and disadvantages [10].
